# Reducing stunting in Bhutan: an achievable national goal†

**DOI:** 10.1111/mcn.12287

**Published:** 2016-05-17

**Authors:** Laigden Dzed, Kinley Wangmo

**Affiliations:** ^1^ Department of Public Health Ministry of Health Thimphu Bhutan

The kingdom of Bhutan is a tiny country, landlocked between the two Asian giants of India and China, and has a population of about 762 864 (Bhutan at a glance 2014). Despite the very small size, the country has made remarkable progress towards modern development since initiating planned development in the 1960s. Bhutan has also experienced rapid progress in many of the key determinants of nutrition and health.

The life expectancy of the Bhutanese has been increasing steadily over the years, currently standing at 68 years (Statistical Yearbook of Bhutan 2014). The country's infant mortality rate has declined to 30 per 1000 live births down from 90 per 1000 in 1990 (National Health Survey (NHS) 2012). The 2010 Multiple Indicator Survey showed the prevalence of stunting in children under 5 years to be 33.5%, indicating a 24% decline from 1986 levels (Bhutan Multiple Indicator Survey).

## Stunting in Bhutan

Stunting or poor linear growth, in young children, is caused by multiple determinants, including antenatal, intra‐uterine and postnatal malnutrition (Waterlow 1994). According to the 2010 Multiple Indicator Survey, the prevalence of stunting is higher in the poorer districts, particularly, in the east of the country. In that region, 43% of the children under five are stunted compared with 31% in the West region and 28% in the Central region. In the poorest wealth quintile, the prevalence of stunting is 41% compared with 21% in the richest quintile (Bhutan Multiple Indicator Survey (BMIS) (2011)).

A situation analysis of nutrition in Bhutan points to the following as major determinants of stunting: diarrheal diseases, high parasite loads in parts of the country and high prevalence of Helicobacter pylori infections. Diseases related to environmental and personal hygiene and the poor nutrition and care of women before and during pregnancy were also identified as risk factors for stunting (Atwood *et al.* 2014).

## Improving linear growth in Bhutan

The nutrition agenda, including stunting reduction, has become a national priority in Bhutan. In the current 5‐year plan (which ends at 2018), stunting reduction is one of the key performance indicators at the national level, which needs to be achieved by the end of the planned period (Eleventh Five Year Plan (2013–2018) 2013). Specific interventions undertaken by Bhutan in the past decade to reduce undernutrition include a focus on optimizing infant and young child feeding practices, supplementation for children, adolescents and pregnant and lactating women with essential micronutrients and the establishment of treatment and rehabilitation centres in health facilities for severely malnourished children.

Although the interventions undertaken by Bhutan are time‐tested and known to have worked in other countries, there is still room for improvement. Strengthening the existing programmes, and continuing to advocate to high level decision makers, to ensure that nutrition remains the top agenda, is important. Coordinating with multiple sectors for targeted funding to improve the coverage and quality of maternal and child nutrition services will also help the nation's effort to improve linear growth in children. These programmes should be undertaken in the backdrop of broader socio‐economic improvements if stunting is to be reduced in the country.

## Role of maternal nutrition, child feeding, and household sanitation

Stunting really begins at conception, and maternal nutrition plays an important role in the development and growth of the fetus. Poor maternal nutrition has been related to intrauterine growth retardation and adverse birth outcomes (Villar *et al.* 2003). In Bhutan, many interventions to promote maternal health and fetal growth are delivered through the health system. It is recommended for pregnant women to come for a minimum of eight antenatal care visits (ANC). An in‐depth analysis of determinants of child stunting in Bhutan found that, after controlling for many other variables, children whose mothers received three or fewer ANC visits during the last pregnancy had 31% higher odds of being stunted, while children whose mother did not receive any ANC visits had 51% higher odds of being stunted (Aguayo *et al.* 2015). ANC promotes optimal nutrition and delivers specific interventions, such as anaemia prophylaxis and treatment, de‐worming prophylaxis, monitoring of fetal growth and assessment of the mothers health. However, during the last nationally represented survey, only 26% of pregnant women in Bhutan were found to have had eight ANC visits.

Adequate nutrition during infancy and early childhood is essential to ensure the growth, health and development of children to their full potential. In terms of infant and young child feeding practices, Bhutan has not been faring well. Only 49% of children under 6 months are exclusively breastfed, while 63% of the infants between the ages of 6 and 23 months receive the minimum frequency of complementary feeds. The role of infant and young child feeding is particularly important for Bhutan as children who were not fed complementary foods at 6–8 months had about threefold higher odds of being severely stunted than children who were fed complementary foods (Aguayo *et al.* 2015).

In Bhutan, 96% of the population use improved sources of drinking water, while 3% of the population practice open defecation. However, only 58% of the population has access to improved sanitation. The lack of safe drinking water and basic sanitation are known to undermine efforts to combat poverty and diseases. The 2010 Multiple Indicator Survey found the highest stunting rates in the eastern region and among people from the poorest wealth quintile, which was also where the sanitation coverage was the lowest.

## Future of stunting in Bhutan

Gaps are still present in child feeding, maternal nutrition and household sanitation in Bhutan, but many encouraging changes are also taking place to cover these shortcomings. Every mother in Bhutan is now tracked to ensure that she benefits from the required number of ANC visits; discussions to extend maternity benefits have started among the highest levels of decision makers and advocacy campaigns to improve breastfeeding, and complementary feeding are ongoing. Efforts are also underway to improve water and household sanitation in the country.

In addition to ongoing child feeding, maternal nutrition and household sanitation interventions, Bhutan has also been making significant strides in socio‐economic development. Between 2007 and 2012, the percentage of poor halved to 12%, and Bhutan has nearly ended extreme poverty (a low of 2% in 2012) (Bhutan Poverty Assessment 2014). The average economic growth between 2009 and 2013 was 6.7 % (National Accounts Statistics 2014), which is a very respectable number for a small donor dependent country (12).

Bhutan's interventions are similar to the interventions undertaken by Brazil, which were very successful in reducing stunting levels through vast targeted funding to improve access to maternal and child health and nutrition services coupled with broad social, economic and political changes (Requejo 2015). With the set of interventions that are already in place and those that are being scaled up, there is no reason why Bhutan cannot replicate or even exceed the Brazilian success.

## Source of funding

United Nations Children's Fund (UNICEF).

## Conflicts of interest

The authors declare that they have no conflicts of interest.

## Authorship responsibilities

LD did the research, planning, and manuscript writing. KD did the review and manuscript writing. All authors have read and approved the final manuscript. The opinions expressed in this paper are those of the authors and do not necessarily represent an official position of the organizations they are affiliated with.
